# Over-expression of microRNA171 affects phase transitions and floral meristem determinancy in barley

**DOI:** 10.1186/1471-2229-13-6

**Published:** 2013-01-07

**Authors:** Julien Curaba, Mark Talbot, Zhongyi Li, Chris Helliwell

**Affiliations:** 1 CSIRO Plant Industry, GPO Box 1600, Canberra, ACT 2601, Australia; 2 Current address: School of Agriculture and Food Systems, University of Melbourne, Parkville, Victoria, 3010, Australia

**Keywords:** Barley, miR171, Scarecrow-like, Phase change, Meristems, Flowering time

## Abstract

**Background:**

The transitions from juvenile to adult and adult to reproductive phases of growth are important stages in the life cycle of plants. The regulators of these transitions include miRNAs, in particular miR156 and miR172 which are part of a regulatory module conserved across the angiosperms. In Arabidopsis miR171 represses differentiation of axillary meristems by repressing expression of SCARECROW-LIKE(SCL) transcription factors, however the role of miR171 has not been examined in other plants.

**Results:**

To investigate the roles of mir171 and its target genes in a monocot, the *Hvu pri-miR171a* was over-expressed in barley (*Hordeum vulgare* L. cv. Golden promise) leading to reduced expression of at least one *HvSCL* gene. The resulting transgenic plants displayed a pleiotropic phenotype which included branching defects, an increased number of short vegetative phytomers and late flowering. These phenotypes appear to be the consequence of changes in the organisation of the shoot meristem. In addition, the data show that miR171 over-expression alters the vegetative to reproductive phase transition by activating the miR156 pathway and repressing the expression of the *TRD (THIRD OUTER GLUME)* and *HvPLA1 (Plastochron1)* genes.

**Conclusions:**

Our data suggest that some of the roles of miR171 and its target genes that have been determined in Arabidopsis are conserved in barley and that they have additional functions in barley including activation of the miR156 pathway.

## Background

During their life cycle, flowering plants undergo three developmental phases, the juvenile and adult vegetative stages and a reproductive phase. During the last decade it has become clear that microRNAs (miRNAs) are important regulators of transitions between these phases. miRNAs are small regulatory RNA molecules which trigger the post-transcriptional repression of target genes through a base-pairing mechanism [1]. There are some 20 miRNA families that are highly conserved in flowering plants. Many of these conserved miRNA families control crucial developmental processes through the down-regulation of conserved transcription factor-encoding genes.

The miRNAs with the best-defined roles in regulating phase changes in the shoot meristem are miR156 and miR172 which form a regulatory module that is widely conserved in plants. In Arabidopsis, rice and maize, miR156 regulates shoot branching, leaf initiation and juvenile-to-adult phase transition through the down-regulation of several *SPL* genes [2-9]. In grass plants, over-expression of miR156 promotes vegetative branching producing an increased number of tillers but inhibits inflorescence branching resulting in a reduced number of spikelets [2-5,9]. Detailed analysis of miR156 function in the maize inflorescence has shown that it has a role in establishing axillary meristem boundaries by spatially restricting expression of the *SPL* gene *Tasselsheath4 (TSH4)* to the bract. This leads to the allocation of more cells to the outgrowth of spikelet meristems at the expense of leaf initiation [3,10]. Recent studies have demonstrated cross talk between the miR156 and miR172 pathways, which appear to have conserved roles in coordinating the timing of vegetative phase changes and competency to flower in all angiosperms [11]. During vegetative shoot development in Arabidopsis and maize, miR156 expression gradually decreases and is inversely correlated with that of miR172 [2,12,13]. Over-expression of miR156 leads to a decrease in miR172 abundance in young shoots [2,13]. In Arabidopsis miR156 down-regulates *AtSPL9/10* which in turn directly activates the transcription of *MIR172b*[13]. The *AtSPL3/4/5* genes are regulated both transcriptionally and post-transcriptionally by miR172 [14] and miR156, [15], respectively. In maize, miR172 is thought to repress the maintenance of juvenile traits and promote the onset of reproductive development by down-regulating *Glossy15 (GL15)*[16]. miR172 expression reaches a maximum in reproductive shoots and promotes floral initiation and flower development by repressing expression of *AP2-Like (AP2L)* genes [12,13,17-23]. In maize and barley, loss of miR172 function promotes the formation of ectopic undifferentiated branches in the place of spikelet meristems (SMs) which in barley eventually lead to the formation of a nascent spike [12,24]. In barley miR172 has also been shown to be responsible for the cleistogamy phenotype by repressing an *AP2-like* gene, *Cleistogamy1 (Cly1)*, in the lodicules, preventing the outgrowth and opening of the floret [25].

miR171 is a well conserved miRNA family known to regulate members of the SCARECROW-LIKE (SCL) transcription factor family. The SCLs belong to a subclass of the highly conserved GRAS family composed of homologs of GAI, RGA and SCR [26]. Among other functions, GRAS proteins are known to be involved in GA responses controlling flowering and regulating apical meristem development [26,27]. In Arabidopsis, there are three *MIR171* genes (a, b and c) which are predicted to regulate three *SCL6* genes (*SCLII, III, IV,* also known as *HAIRY MERISTEM* (*HAM*) and *LOST MERISTEMS* (*LOM*), [28-30]. The expression domains of the *miR171* family members and the *SCL6-II/III* mRNAs overlap, suggesting a redundant function for both miRNA and target mRNAs [30-32]. miR171a is most highly expressed in the inflorescence where it regulates *SCL6-III* and *SCL6-IV* expression through mRNA cleavage [29,33]. Arabidopsis plants over-expressing miR171c (OE171c) and the triple *scl6* mutant show similar pleiotropic phenotypes, including altered shoot branching, plant height, chlorophyll accumulation, primary root elongation, flower structure, leaf shape and patterning, indicating that miR171 acts mainly by down-regulating *SCL6* genes to control a wide range of developmental processes during shoot development [32]. The reduced branching phenotype observed in OE171c plants can be rescued by the expression of a miR171c-resistant version of *SCL6-II, III*, or *IV*[32], suggesting that the three targets have partially redundant function. *SCL6-IV,* which is expressed at the boundary between the axillary meristem and the SAM, seems to have a different function to the two other *SCL6* genes, being linked to branching but not SAM maintenance [30,32]. A more detailed analysis of *SCL6-II/III* showed that these genes are expressed in the peripheral zone and vascular tissues of the SAM and promote the maintenance of the pool of meristematic cells and the differentiation of the axillary meristem [28,30].

Although studies in Arabidopsis have revealed important roles for miR171 and its SCL targets, their roles in monocot plants are unknown. This study investigated the role of the miR171-SCL module in controlling plant architecture in barley (*Hordeum vulgare* L. cv Golden promise). Over-expression of miR171 affects expression of meristem identity genes suggesting a conservation of the role identified in Arabidopsis. In addition a delay in the transition to reproductive growth involving miR156 was observed which suggests that there are monocot specific functions for miR171 and its target genes.

## Results and discussion

### Conserved molecular functions of miR171 in barley

In barley two mature miR171 sequences (hvu-miR171a/b) have been identified [34] which differ by one central nucleotide. The only miRNA precursor identified is that for hvu-miR171a (Additional file 1). There are nine rice, fourteen maize and four Brachypodium miR171 family members in miRBase, indicating that barley is likely to have additional *miR171* genes.

**Figure 1:**
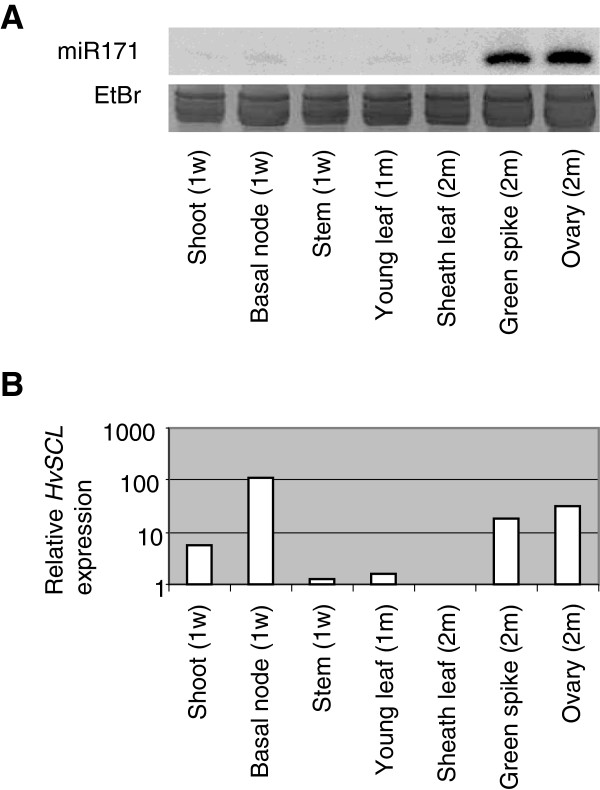
**Expression profile of hvu-miR171 and *HvSCL*. (A)** Northern blot using a hvu-miR171a antisense probe. The tissues were harvested from different stages of development and the age in weeks (w) or months (m) is indicated in brackets. **(B)** RT-qPCR showing the relative abundance of *HvSCL* mRNA in the same tissues used for the northern blot in **(A)**, using the level in the sheath leaf as reference.

The abundance of miR171 was examined in various tissues and it was found to be predominantly expressed in reproductive tissues (Figure 1A). These data correlate with previous observations in Arabidopsis where miR171 mostly accumulates in reproductive organs [29,31,33]. The psRNAtarget server ( http://plantgrn.noble.org/psRNATarget) was used to search for potential targets of hvu-miR171a and b amongst a recently assembled full-length cDNA dataset from barley [35]. This analysis identified 11 potential target ESTs predicted to be miR171-regulated by cleavage or translational repression using a mismatch score of 4.0 or less (Additional file 2). Of the target mRNAs with the lowest mismatch scores (1.5 or less), three (AK368048, AK371946 and AK364580) encode SCL6-like proteins and are all predicted to be regulated by miR171 cleavage. The fourth potential miR171 target (AK362896) encodes a protein of unknown function and is predicted to be regulated by translational repression. We searched degradome data generated from developing barley seed [36] for evidence of cleavage of these four mRNAs and found evidence for cleavage of the three *SCL6-like* mRNAs but not AK362896 (Additional file 3: AK364580 and AK368048 are identical at the miR171 cleavage site so cannot be distinguished in the degradome data). The degradome data for AK371946 had the greatest number of reads 2 bp upstream of the predicted cleavage site however RLM-5^′^ RACE showed that the predicted miR171 cleavage site was the most abundant (Additional file 3).

### Mis-expression of miR171 in barley leads to pleiotropic phenotypes

**Figure 2:**
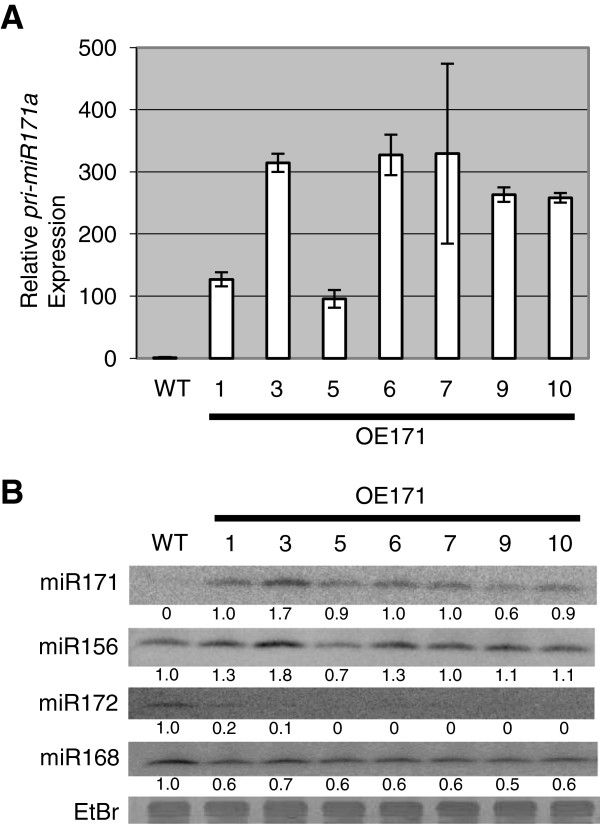
**Expression of *pri-miR171a *and miR171 in OE171 transgenic lines. (A)** RT-qPCR showing the abundance of *pri-miR171a* mRNA in T_0_ leaves of OE171 lines (numbered) relative to WT (set as 1.0). **(B)** Northern blot showing the level of different mature miRNA sequences in leaves of T_0_ OE171 lines and WT. miR168 was used as loading control.

To study the role of miR171 and its targets in barley, *pri-miR171a* was over-expressed under control of the maize ubiquitin promoter. Seven independent transgenic lines were obtained with at least one hundred fold over-expression of *pri-miR171a* compared to wild-type (Figure 2A). A corresponding increase in the abundance of mature miR171 was also detected in all the transgenic lines (Figure 2B). The T_o_ miR171 over-expressing plants (OE171) showed a pleiotropic phenotype with altered shoot architecture and delayed flowering. For lines where we were able to obtain seed, these phenotypes were confirmed in the T_1_ generation. In our subsequent analyses, T1 lines 1, 6 and 9 were used; these had similar phenotypes and expression of the mature miR171. T_1_ OE171 plants were selected based on the presence of the transgene and grown under long day (LD) and short day (SD) conditions with 16 and 10 hour photoperiods, respectively.

**Figure 3:**
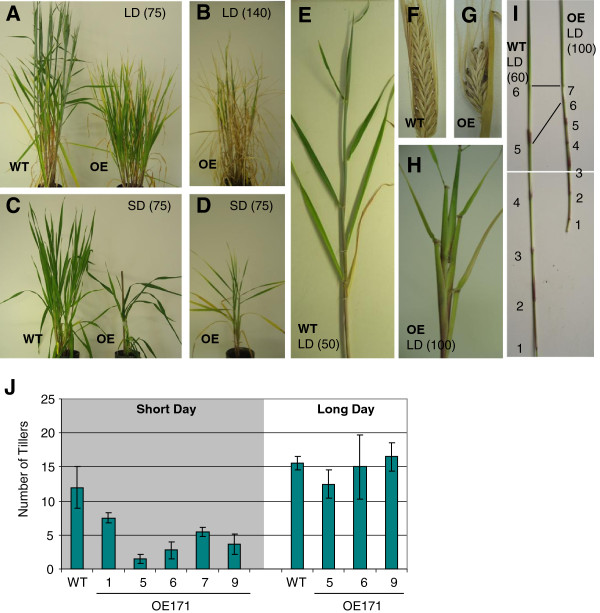
**OE171 affects barley shoot architecture.** T_1_ OE171 plants (OE) and WT grown under long day (LD) or short day (SD) conditions **(A-E)**. The plant age is indicated in Days Post Germination (DPG). **(F-G)** Dry spikes. **(I)** WT and OE171 culms without the leaves showing the nodes numbered from the crown. **(J)** Number of emerging tillers on 2 month-old plants grown under SD and LD. 2 to 4 plants were observed for each transgenic line.

Under LD conditions the first wild-type (WT) stems stopped producing leaves and started to elongate at 30 DPG (Days Post Germination) whereas OE171 plants at the same age continued to produce new leaves, indicating a juvenile-to-adult transition delay. The internodes of OE171 plants started to extend after 75 DPG, while WT plants were already flowering (Figure 3A, Additional file 4). OE171 plants were extremely late flowering with the first spike starting to flower after 140 DPG (Figure 3B). At this stage the stems were fully elongated and OE171 plants were dwarfed compared to WT at the same stage of development (Figure 3A, B). The dwarfism was due to reduced internode length, resulting in a compaction of the leaves wrapped around the inflorescence (Figure 3E, H, I). OE171 produced on average one or two additional visible nodes, with a decreasing internode length towards the top of the stem (Figure 3I). The transgenic plants eventually developed short and partially sterile spikes (Figure 3F, G). A similar phenotype was observed under SD conditions where OE171 showed an increased number of phytomers, reduced internode length (Figure 3C, D) and a delay in the juvenile-to-adult transition compared to the WT grown under the same conditions (Additional file 4). In addition, OE171 plants grown under SD produced fewer tillers (on average 4 tillers compared to 12) than the WT plants (Figure 3C, D, J, Additional file 5) and never developed any fertile spikes.

**Figure 4:**
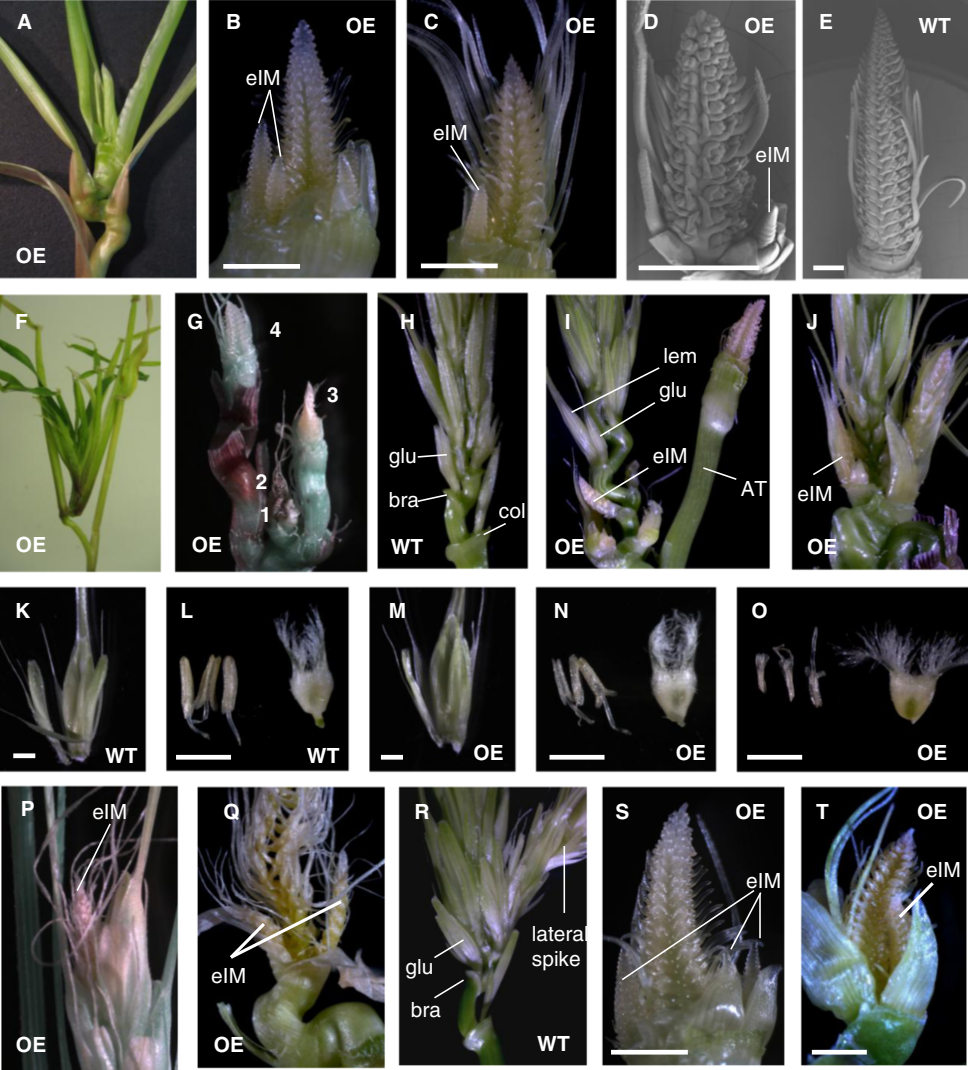
**OE171 affects floral meristem differentiation and pollen development.** T1 plants of OE171 **(OE)** and WT grown under LD **(A-Q)** and SD **(R-T)** conditions. **(A)** OE171 Shoot with leaves manually opened. **(B-C)** Young OE171 inflorescence at 100DPG showing ectopic inflorescence meristems (eIM). **(D-E)** Scanning electron microscopy of OE171 and WT inflorescences at 100DPG and 50DPG respectively. **(F)** Condensed leaf structure on the apical shoot of an OE171 plant. **(G)** Aerial tillers producing multiple inflorescence meristems (numbered 1 to 4) revealed after removing the leaves from (F). **(H-J)** Base of green spikes at flowering time showing the first rachis phytomers; glumes (glu), bract (bra), collar (col), lemma-awn (lem), aerial tiller **(AT)** and ectopic inflorescence meristems. **(K)** WT floret, **(L)** WT anthers and ovary, **(M)** OE171 floret, **(N-O)** OE171 anthers and ovary. **(P)** Ectopic inflorescence meristems emerging from the top of a OE171 mature spike. **(Q)** OE171 inflorescence dried out at 150DPG. **(R)** Base of a WT green spikes grown under SD showing an ectopic lateral spike. **(S-T)** Young inflorescence of OE171 at 120DPG and 150DPG (T). Scale bars represent 1 mm.

A closer examination of the shoot apex of OE171 plants grown under LD showed an accumulation of phytomers with a progressive reduction of bending at the internode. These eventually form a compact structure of joined nodes with a series of leaves that wrap around the inflorescence (Figure 4A). The main inflorescences, as well as the resulting spikes (Figure 3G), were shorter with a reduced number of spikelet meristems compared to the wild-type (Figure 4D, E). The development of the spikelet meristems into floral organs was delayed, but their morphology and phyllotaxy on the inflorescence meristem appeared normal (Figure 4B to E). At the base of each OE171 inflorescence one to at least four indeterminate meristems which formed new inflorescence meristems were observed (Figure 4B, C, D). These ectopic inflorescence meristems were sometimes preceded by the formation of short aerial tillers producing additional leaves in a condensed structure at the top of the culm (Figure 4F, G). The formation of a new branch appeared to be at the expense of the adjoining inflorescences which stopped developing and dried out (Figure 4G). At the base of the WT spike at flowering stage, the floral structure on the first rachilla phytomer was restricted to a bract (instead of a lemma-awn) and a pair of glumes (Figure 4H). In comparison, OE171 spikes at the same developmental stage possess several phytomers with intermediate culm/rachis characteristics at the base of the inflorescence (Figure 4I, J). Internode lengths were intermediate between those of culm and rachis phytomers and indeterminate inflorescence meristems emerged from the axillary buds instead of spikelet meristems (Figure 4I). These secondary inflorescence meristems eventually produced lemma-awn structures but never grew and formed a fully developed floret (Figure 4J). Meanwhile, the main inflorescence produced several WT-like florets in the middle of the spike but only a few were fertile (Figure 4K, M). OE171 anthers were smaller and translucent in comparison to the WT, suggesting a pollen development defect that could explain the partial sterility phenotype (Figure 4L, N, O, 3G). In addition, the terminal branch structure of the spike sometimes gave rise to an ectopic inflorescence meristem (Figure 4P). In the strongest lines, the developmental delay was such that the spike did not form any floral organs and dried out (Figure 4Q). The OE171-3 T_0_ plant had the highest miR171 expression and never flowered. It developed tillers with an extremely disorganized inflorescence with a phyllotaxy defect of the spikelet meristems which never transitioned to floral meristems (Additional file 5). Under SD conditions WT plants sometimes developed one ectopic inflorescence meristem at the base of the primary inflorescence which eventually formed a fertile lateral spike (Figure 4R). In comparison, the inflorescences of OE171 plants developed several ectopic inflorescence meristems, as observed in long day conditions (Figure 4S), but the developmental delay was stronger and the spikes dried out before reaching the flowering stage (Figure 4T).

These observations suggest that barley miR171 can regulate axillary meristem development and the timing of the juvenile to adult phase transtition. In Arabidopsis, miR171 acts mainly through the down-regulation of the *SCL6-II/III/IV* genes; over-expression of miR171 or loss of *SCL6* function represses axillary meristem differentiation resulting in reduced shoot branching [30,32], and eventually a complete developmental arrest under SD conditions [30]. This phenotype correlates with the observations here and suggests that miR171 regulates shoot branching through a conserved mechanism between monocots and dicots. However, in contrast to the OE171 phenotype in barley, OE171 in Arabidopsis also promotes stem elongation [32], represses leaf initiation [30] and only slightly delays flowering [28], suggesting that the roles of miR171 and its targets evolved differently for these processes in barley and Arabidopsis.

### Mis-regulation of gene expression in OE171 barley plants

**Figure 5:**
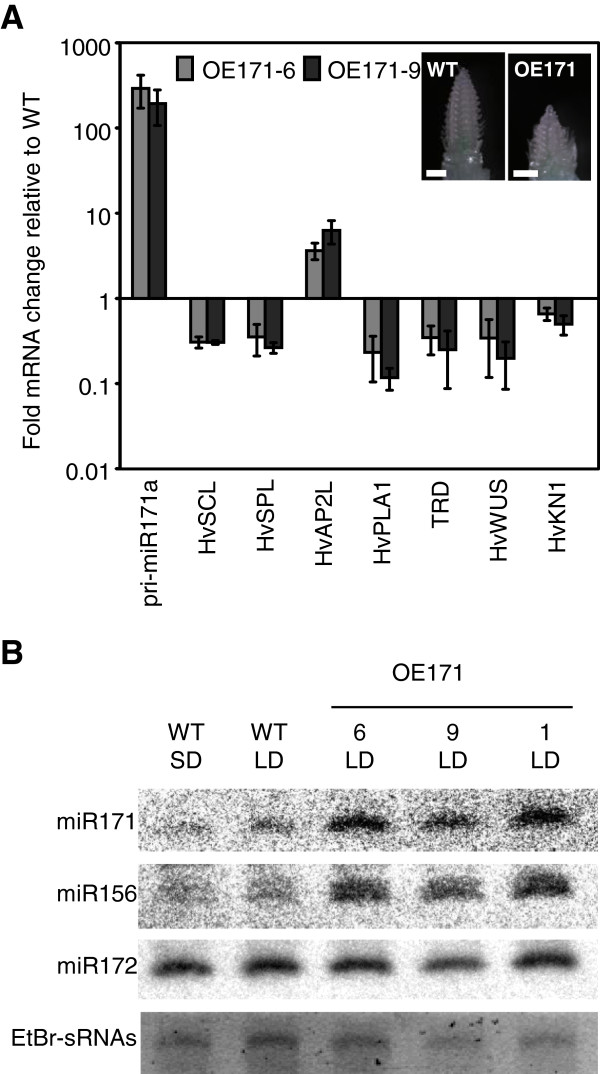
**Hvu-miR171-SCL molecular pathway. (A)** RT-qPCR showing the mRNA abundance of different genes of interest in young inflorescence of two independent OE171 lines relative to WT at similar developmental stage (as shown by the SAM pictures on the top right corner; scale bars = 1mm), grown under LD. **(B)** Northern blot showing the level of different mature miRNA sequences in young inflorescence of three independent OE171 lines grown in LD and WT plants grown in SD and LD. Ethidium bromide staining of the small RNAs was used as loading control (SAM, shoot apical meristem; AM, axillary meristem).

In both barley and Arabidopsis, OE171 affects shoot branching [32]. In Arabidopsis, the miR171 family acts mainly through the down-regulation of three *SCL6* genes *(SCL6-II/III/IV)*[32]. As OE171 disrupts meristem function we extracted RNA from young inflorescence meristem tissues of T_1_ OE171 transgenic lines and WT plants grown under short day conditions to quantify the abundance of miR171 and its targets. All transgenic lines showed an over-accumulation of the *pri-miR171a* transcripts and mature miR171 sequences (Figure 5A, B). We were unable to detect AK364580, AK362896 and AK368048 in meristem tissues by RT-qPCR (data not shown). AK371946 was expressed in wildtype and showed reduced levels in OE171 plants (Figure 5A) suggesting that this was the main target down-regulated by miR171 over-expression in the shoot apex. However it is possible that other target genes with higher mismatch scores are affected in OE171 plants and that the other *SCL6* genes are targeted by miR171 outside of the meristem tissues that we have focused on. For simplicity we refer to AK371946 as *HvSCL* from here onwards.

We determined the tissue specificity of *HvSCL* expression by RT-qPCR (Figure 1B). *HvSCL* is expressed in the shoot, basal node, green spike and ovary. The strong expression of *HvSCL* in green spike and ovary overlaps with the highest miR171 abundance, at least at the tissue level, suggesting that miR171 acts to dampen *HvSCL* expression rather than restrict it [37]. These data correlate with previous observations in Arabidopsis where both miR171 and *SCL* target mRNAs are predominantly detected in the same tissues [33].

RNA *in situ* hybridization of Arabidopsis shoot meristem tissues shows that SCL6-II/III are mostly expressed in the peripheral zone and the vascular strands of developing axillary buds where they promote cell differentiation and help maintain the polar organization of the SAM [30]. The maintenance of the SAM requires the expression of *WUSCHEL (WUS)* and the knotted-like gene *SHOOT MERISTEMLESS (STM)* to prevent the differentiation of the meristematic cells located in the central zone. In the double mutant *scl6-II scl6-III* grown under short day conditions, the spatial expression patterns of *WUS* and *STM* are altered [30]. A *WUS*-related homeobox gene, *HvWUS* (U21_34817), and a class I *Knotted-like* gene, *HvKN1* (TC279468) were identified in barley EST databases. Quantification of their relative expression in the inflorescences of OE171 plants shows a reduction for both genes compared to the WT (Figure 5A). This effect may reflect a disorganization of the SAM and loss of maintenance of meristem cells, which correlates with miR171 function in Arabidopsis and could explain the reduction of shoot branching observed in barley OE171.

The OE171 phenotype resembles that of OE156 in maize [2,12]. In maize, miR156 controls plant architecture by regulating at least 2 SPL genes, *Teosinte Glume Architecture1 (TGA1)* and *Tasselsheath4 (TSH4)*[10,12]. The dominant *Corngrass1 (Cg1)* mutant, in which zma-miR156b/c is over-expressed, shows an extended vegetative program characterized by an increased number of leaves and the development of large bract leaves in the inflorescences [2]. Over-expression of zma-miR156b/c also causes the reduction of zma-miR172 abundance which is known to control juvenile-to-adult phase transition through the down-regulation of *Glossy15 (GL15)*[16] and spikelet meristem determinancy through the repression of *IDS1* and *SID1*[12,19]. In barley, the phenotype associated with loss of miR172 function is similar to the OE171 plants, blocking spikelet meristem determinacy and exhibiting ectopic branch structures from the base and the end of the spike [24] (Figure 4A). These phenotypic similarities suggest that miR171 is linked to the miR156-miR172 pathway in barley.

The effect of OE171 on miR156 and miR172 expression was examined using inflorescence tissue from T_1_ OE171 and WT plants grown under LD conditions (Figure 5B). miR156 abundance was higher in OE171 lines but no change in miR172 expression was detected. Using the same tissues, we quantified the relative abundance of two barley mRNAs, coding for an *SPL* gene predicted to be targeted by miR156, *HvSPL* (U21_18637) and a potential target of miR172, *HvAP2L* (U21_18652) (Figure 5A). As expected, *HvSPL* was down-regulated in OE171, reinforcing the hypothesis that miR171 acts at least partially through the up-regulation of miR156. Surprisingly, *HvAP2L* was up-regulated in OE171, suggesting that miR171 regulates *HvAP2L* expression independently from miR172. This last observation correlates with previous work in Arabidopsis reporting that *TOE3* (an *AP2-like* gene containing a miR172 binding site) expression gradually increases along with miR171 abundance in the developing shoot [20]. Additionally, AP2 was recently shown to directly activate *MIR156e* expression in Arabidopsis [38], suggesting that the up-regulation of *HvAP2L* in OE171 could be responsible for the increasing abundance of miR156.

The increased accumulation of miR156 in OE171 did not cause a reduction of miR172 abundance in contrast with previous observations in Arabidopsis and maize [2,13]. However, observations by Jung et al. [14] showed that OE156 driven by an inducible promoter in Arabidopsis did not noticeably affect miR172 abundance. The authors proposed that miR156 principally affects miR172 during the early vegetative stage by repressing *MIR172b* expression and that the increasing miR172 abundance during the late vegetative stage is driven by other *MIR172* genes independent of miR156. This correlates with the observation that miR172 abundance continues to increase during later vegetative stages while the miR156 level stays at a constant minimum level [13,14]. In agreement with this hypothesis, a decrease in miR172 expression was detected in the young leaves of OE171 T_0_ plants which over-accumulated miR156 (Figure 2B). Maize OE156 plants did not show the same flowering defect as for loss of miR172 function [2,12], suggesting that the function of miR172 in the inflorescence is independent of miR156.

The phenotype of the barley OE171 plants was also reminiscent of mutants in maize, rice and barley genes that affect the rate of leaf initiation. We did not observe any clear changes in the leaf initiation rate in OE171 (Additional file 6) but similarities in other phenotypes led us to examine these genes in OE171 plants. In Arabidopsis the leaf initiation rate is likely to be controlled by two independent pathways, one involving miR156-*SPLs* and the other involving two orthologs of the rice *PLASTOCHRON1 (PLA1)* gene which encode cytochrome P450 enzymes [39,40]. In rice, loss of *PLA1* function causes an extension of the vegetative program characterized by an increased number of short vegetative phytomers and the production of vegetative shoots (with the emergence of new leaves) instead of primary rachis branches [41], a phenotype that closely resembles OE171 in barley. More recently it has been shown that *PLA1* expression is under control of the transcription factor *NECK LEAF 1 (NL1)*, which when mutated causes a similar phenotype to that of the *pla1* mutant [42]. *NL1* is orthologous to *Third Outer Glume (TRD)* in barley and *Tasselsheath-1 (TSH-1)* in maize. In both *trd* and *tsh-1* mutants the repression of bract growth is suppressed, producing leaves that sheath the inflorescence and a reduction in the number of inflorescence branches [43]. Therefore the regulation of *TRD* and *PLA1* by miR171 in barley was investigated. *TRD* was previously cloned using degenerate PCR (GU722206, [43] and one barley EST homolog to *PLA1* was found, *HvPLA1* (U21_15786). The mRNA abundance for both genes was significantly reduced in the inflorescences of OE171 compared to the WT (Figure 5A). These results suggest that miR171 coordinates vegetative phase changes in barley through the regulation of two potentially distinct pathways. It is proposed that miR171 acts up-stream of miR156 and *TRD* which in turn may regulate Hv*PLA1* expression as was observed for their orthologs in rice [44].

## Conclusion

Overall, the data presented here suggest roles for miR171 and its targets in regulating shoot development in barley. Over-expression of miR171 results in an extended vegetative phase characterised by an increased number of leaves and the initiation of indeterminate axillary meristems instead of spikelet meristems. Additionally, OE171 plants have a reduced number of tillers emerging from the axillary meristems of the crown (under SD conditions) and a delay in the differentiation of spikelet meristems into floral organs. A model is proposed in which miR171 is an upstream regulator that coordinates the timing of shoot development in barley through three independent pathways. First, the results, together with current knowledge from Arabidopsis, suggest that miR171 affects meristem maintenance and axillary meristem differentiation in barley through the down-regulation of *HvSCL*[28,30], and consequently affects the expression of meristem specific genes such as the homologs of *WUS* and *KN1* analysed in this study. Secondly, miR171 could repress vegetative phase transitions in barley through the upregulation of miR156, a known regulator of the transition from juvenile to adult phases across the angiosperms [2,6-9,13,45]. Interestingly, OE171 and OE156 in Arabidopsis show opposite effects on leaf initiation [30,32,40], suggesting that the possible connection between the miR171 and miR156 pathways may be monocotyledon specific. Thirdly, miR171 promotes vegetative traits in barley through a secondary pathway, independent from miR156 [40], that involves *TRD* and *HvPLA1*[42,43]. These apparent additional roles for miR171 and its targets in barley shoot development may represent an important evolutionary difference between monocot and dicot plants.

## Methods

### Vector construction

The sequence corresponding to *pri-miR171a* was amplified using the primers MIR171-1F/2R (Additional file 7) and cloned into the pENTR/D topo vector (Invitrogen). The construct was then recombined (according to the manufacturer’s instructions) into the *Agrobacterium* binary vector pWBVec8 [46] modified to contain a Gateway cassette down-stream of the Ubiquitin promoter. The construct was then introduced into the *Agrobacterium* strain AGL1 which was used to transform Golden Promise barley plants.

### Plant transformation and growing conditions

The transformation was performed as described [47]. In brief, barley plants were grown at 12°C / 16h light and immature grains were harvested at about DPA20 (when the embryo turned from translucent to white and the scutellum is about 2mm in diameter). After sterilization of the grains, the embryos were isolated and the axis was removed. Dissected scutella (approximately 150) were put in contact with a fresh culture of AGL1-MIR171a for 3 days on antibiotic-free media. Transformed calli were selected on hygromycin containing media. The resulting T_0_ plants were transferred into soil and grown in naturally lit phytotron glasshouses. T_1_ plants were selected for the presence of the transgene and grown with air temperature set at 17°C/9°C day/night cycle under long day (LD) and short day (SD) conditions with 16 and 10 hours of day light, respectively.

### Quantitative RT-PCR

RT-PCR reactions were performed as described [21]. In brief, total RNA was extracted using TRIzol reagent (Invitrogen) and 4μg was used for first-strand cDNA synthesis using Oligo(dT) primers and the Super-Script III RT kit (Invitrogen). PCRs were performed according to the manufacturer on an ABI 7900 HT Fast Real-Time PCR System (Applied Biosystems). 1μl of 1:10 diluted template cDNA was used in a 10μl reaction. The amplification program was: 15″ at 95°C, (15″ at 95°C, 30″ at 60°C, 30″ at 72°C) for 35 cycles, followed by a thermal denaturing step. Relative transcript levels were calculated with the ΔΔCt method (Applied Biosystems) using the *ACTIN* gene as a reference. Each value presented in the histograms represents an average from three independent replicates plus/minus Standard Deviation. Forward and reverse primer sequences are presented in Additional file 7.

### RNA gel blot analysis

40 μg of total RNA, extracted using TRIzol reagent (Invitrogen) and separated on a denaturing 15% polyacryamide gel containing 7M urea at 120V for 2hr. RNA loading was based on spectroscopic measurement and confirmed by ethidium bromide staining of the gel prior to transfer. RNA was electrophoretically transferred to Zeta-probe GT membrane (BioRad) at 40V for 90 min and fixed by UV crosslinking. The membrane was first incubated in hybridization buffer [Na2PO4-pH7.2 125 mM, NaCl 250mM, SDS 7%, formamide 50%] for 4h at 42°C and then incubated in the presence of 32P-end-labeled oligonucleotide probes at 42°C overnight. The membrane was washed in [2× SSC, 0.2% SDS] at 42°C and the radioactivity was detected by phosphorimager. Oligonucleotide probe sequences are presented in Additional file 7.

### RLM-5^′^ RACE

RNA ligase-mediated 5^′^ rapid amplification of cDNA ends (RLM 5^′^-RACE) was performed using the GeneRacer kit (Invitrogen). The manufacturer’s protocol was followed for 5^′^ end analysis with exception of the 5^′^ de-capping step. In brief, total RNA was isolated from 2 week-old shoot tissues and ligated to a 5^′^ end RNA adaptor before being reverse transcribed using an oligo(dT) primer. The PCR reactions were performed using gene specific reverse primers (Additional file 7). The amplicons were gel purified, cloned into the pGEM-T vector (Promega) and sequenced.

## Abbreviations

SCL: Scarecrow-like; miRNA: micro RNA.

## Competing interests

The authors declare no competing financial interests.

## Authors’ contributions

JC designed the study, carried out experiments, analysed data and drafted the manuscript. MT carried out experiments, ZL helped design the study and draft the manuscript. CH designed the study carried out experiments, analysed data and helped draft the manuscript. All authors read and approved the final manuscript.

## Supplementary Material

Additional file 1 **miR171 precursor and mature sequences.** Information retrieved from miRBase (release-17) and Schreiber *et al.*, 2011 [34]. (**A**) *pri-miR171a* secondary structure obtained using MFOLD ( http://mfold.rna.albany.edu), red letters indicate mature miRNA sequence. (**B**) mature miR171 sequences found in *Hordeum vulgare* (hvu), Arabidopsis *thaliana* (Ath) and *Oryza sativa* (Osa). Click here for file

Additional file 2 **Potential targets of hvu-miR171a and b.** psRNAtarget ( http://plantgrn.noble.org/psRNATarget) was used to predict potential targets of hvu-miR171a and b among the full-length barley ESTs assembled by Matsumoto *et al.,* 2011 [35]. The maximum score was set at 4. The column “Activity” refers to the type of inhibition predicted, Cleavage (CL) or Translation inhibition (TR). Click here for file

Additional file 3 **Evidence for miR171 cleavage of HvSCLs and HvSPL.** (**A**) Evidence for cleavage of top four predicted miR171 targets in degradome data from developing barley grains [36] (**B**) RLM-5^′^ RACE on HvSCL (U21_33945). (**C**) RLM-5^′^ RACE on HvSPL (U21_18637). The miRNA binding site is underlined in black and the 5^′^ end of the cleaved mRNA in red. The numbers in green refer to the ratio of 5^′^-RACE clones showing a cleavage at the base indicated by the arrow. The agarose gel on the left shows the band corresponding to the amplified 3^′^ cleavage products. Click here for file

Additional file 4 **Timing of the transition phases during shoot development.** The diagram shows the approximate timing of the transitions from juvenile to adult phases and adult to reproductive phases. The first transition was determined by the moment when the first stem of the plant started to elongate (jointing) and the second transition (flowering) when the first spike reached anthesis. Under our SD condition, OE171 plants did not flower. Click here for file

Additional file 5 **Developmental arrest of OE171-3 (T0).** (**A**) Tiller of the T_0_ plant OE171-3. Scanning electron microscopy of the SAM of a OE171-3 (**B**) and WT (**C**) plants. Ectopic inflorescence meristem (eIM). Scale bars represent 1 mm. Click here for file

Additional file 6 Comparison of vegetative WT and OE171 plants. Representative WT and OE171 plants at 4 weeks old in LD conditions. Click here for file

Additional file 7 Primer and Probe sequences used in this study. Click here for file
