# Supplementary material for: Over-expression of microRNA171 affects phase transitions and floral meristem determinancy in barley
Source: BMC Plant Biol. 2013 Jan 7;13:6. doi: 10.1186/1471-2229-13-6 (PMC3547705; doi:10.1186/1471-2229-13-6)
Supplement: Additional file 1 — miR171 precursor and mature sequences. Information retrieved from miRBase (release-17) and Schreiber et al., 2011 [34]. (A) pri-miR171a secondary structure obtained using MFOLD ( http://mfold.rna.albany.edu), red letters indicate mature miRNA sequence. (B) mature miR171 sequences found in Hordeum vulgare (hvu), Arabidopsis thaliana (Ath) and Oryza sativa (Osa). [file 1471-2229-13-6-S1.pdf]

A. pri-miR171a (U21_38648)

```
        10        20         30         40
U     U         U  A    C    -   A-|    G   GA
 CACUA GAUGUUGGC CG CUCA UCAG ACC  CGCCG AGG  G
 GUGUA CUAUAACCG GC GAGU AGUC UGG  GCGGC UCU  C
C     U         U  C    U    U   CG^    G   AC
 90        80        70        60        50
```

B. Mature miR171 sequences

**hvu-miR171a**	ugauugagccgugccaauauc
**hvu-miR171b**	ugauugagccgcgccaauauc
**ath-miR171a**	ugauugagccgcgccaauauc
ath-miR171b/c	uugagccgugccaauaucacg
**osa-miR171a**	ugauugagccgcgccaauauc
**osa-miR171b-f**	ugauugagccgugccaauauc
osa-miR171g	gaggugagccgagccaauauc
osa-miR171h	gugagccgaaccaauaucacu
osa-miR171i	ggauugagccgcgucaauauc

Additional file 1. miR171 precursor and mature sequences. Information retrieved from miRBase (release-17) and Schreiber *et al.*, 2011 [34]. (A) *pri-miR171a* secondary structure obtained using MFOLD (http://mfold.rna.albany.edu), red letters indicate mature miRNA sequence. (B) mature miR171 sequences found in *Hordeum vulgare* (hvu), Arabidopsis *thaliana* (Ath) and *Oryza sativa* (Osa).
