# Supplementary material for: Over-expression of microRNA171 affects phase transitions and floral meristem determinancy in barley
Source: BMC Plant Biol. 2013 Jan 7;13:6. doi: 10.1186/1471-2229-13-6 (PMC3547705; doi:10.1186/1471-2229-13-6)
Supplement: Additional file 2 — Potential targets of hvu-miR171a and b. psRNAtarget ( http://plantgrn.noble.org/psRNATarget) was used to predict potential targets of hvu-miR171a and b among the full-length barley ESTs assembled by Matsumoto et al., 2011 [35]. The maximum score was set at 4. The column “Activity” refers to the type of inhibition predicted, Cleavage (CL) or Translation inhibition (TR). [file 1471-2229-13-6-S2.pdf]

Genbank ID	Homology	Score	Sequence Alignment	Activity
miR171a targets				
AK368048	SCL	0.0	miRNA 21 CUAUAACCGUGCCGAGUUAGU 1<br>:::::::::::::::::::::<br>Target 932 GAUAUUGGCACGGCUCAAUCA 952	CL
AK371946	SCL	0.5	miRNA 21 CUAUAACCGUGCCGAGUUAGU 1<br>:::::::::.:::::::::::<br>Target 1231 GAUAUUGGCGCGGCUCAAUCA 1251	CL
AK364580	SCL	0.5	miRNA 21 CUAUAACCGUGCCGAGUUAGU 1<br>:::::::::.:::::::::::<br>Target 616 GAUAUUGGCGCGGCUCAAUCA 636	CL
AK362896	Unknown function	1.5	miRNA 21 CUAUAACCGUGCCGAGUUAGU 1<br>:::.::::::.::.:::::::<br>Target 446 GAUGUUGGCAUGGUUCAAUCA 466	CL
AK360395	ATPase	4.0	miRNA 21 CUAUAACCGUGCCGAGUUAGU 1<br>::.:::: .: :::::::::<br>Target 926 UAUGUUGGAGCUGCUCAAUCA 946	TR
AK374235	WD40	4.0	miRNA 21 CUAUAACCGUGCCGAGUUAGU 1<br>::. ::::: ::.:::::::<br>Target 959 CAUGAUGGCAAGGUUCAAUCA 979	CL
AK373446	Unknown function	4.0	miRNA 21 CUAUAACCGUGCCGAGUUAGU 1<br>: ::: :: :::::::::::<br>Target 1247 UAGAUUAGCCCGGCUCAAUCA 1267	CL
AK368182	RNA binding protein	4.0	miRNA 21 CUAUAACCGUGCCGAGUUAGU 1<br>:::::::::::.: .::. :::<br>Target 893 GAUAUUGGCAUGCUUCGCUCA 913	TR
AK252561	Unknown function	4.0	miRNA 21 CUAUAACCGUGCCGAGUUAGU 1<br>::::::: :::::::.:::.<br>Target 96 UUUAUUGGCCCGGCUCGAUCG 116	CL
miR171b targets				
AK371946	SCL	0.0	miRNA 21 CUAUAACCGCGCCGAGUUAGU 1<br>:::::::::::::::::::::<br>Target 1231 GAUAUUGGCGCGGCUCAAUCA 1251	CL
AK364580	SCL	0.0	miRNA 21 CUAUAACCGCGCCGAGUUAGU 1<br>:::::::::::::::::::::<br>Target 616 GAUAUUGGCGCGGCUCAAUCA 636	CL
AK368048	SCL	1.0	miRNA 21 CUAUAACCGCGCCGAGUUAGU 1<br>::::::::: :::::::::::<br>Target 932 GAUAUUGGCACGGCUCAAUCA 952	CL
AK362896	Unknown function	2.5	miRNA 21 CUAUAACCGCGCCGAGUUAGU 1<br>:::.::::: .::.:::::::<br>Target 446 GAUGUUGGCAUGGUUCAAUCA 466	CL
AK360395	ATPase	3.5	miRNA 21 CUAUAACCGCGCCGAGUUAGU 1<br>::.:::: :: :::::::::<br>Target 926 UAUGUUGGAGCUGCUCAAUCA 946	TR
AK248532	E3 ubiquitin ligase	3.5	miRNA 21 CUAUAACCGCGCCGAGUUAGU 1<br>:::::: : :: ::::.::::<br>Target 164 GAUAUUCGAGCUGCUCGAUCA 184	TR
AK366340	Phosphatase associated protein	4.0	miRNA 21 CUAUAACCGCGCCGAGUUAGU 1<br>:::.::: :: ::::::: ::<br>Target 1890 GAUGUUGACGAGGCUCAAGCA 1910	TR
AK373446	Unknown function	4.0	miRNA 21 CUAUAACCGCGCCGAGUUAGU 1<br>: ::: :: :::::::::::<br>Target 1247 UAGAUUAGCCCGGCUCAAUCA 1267	CL
AK252561	Unknown function	4.0	miRNA 21 CUAUAACCGCGCCGAGUUAGU 1<br>::::::: :::::::.:::.<br>Target 96 UUUAUUGGCCCGGCUCGAUCG 116	CL

Additional File 2. Potential targets of hvu-miR171a and b. psRNAtarget (http://plantgrn.noble.org/psRNATarget) was used to predict potential targets of hvu-miR171a and b among the full-length barley ESTs assembled by Matsumoto et al 2011 [35]. The maximum score was set at 4. The column "Activity" refers to the type of inhibition predicted, Cleavage (CL) or Translation inhibition (TR).
