# Supplementary material for: Over-expression of microRNA171 affects phase transitions and floral meristem determinancy in barley
Source: BMC Plant Biol. 2013 Jan 7;13:6. doi: 10.1186/1471-2229-13-6 (PMC3547705; doi:10.1186/1471-2229-13-6)
Supplement: Additional file 3 — Evidence for miR171 cleavage of HvSCLs and HvSPL. (A) Evidence for cleavage of top four predicted miR171 targets in degradome data from developing barley grains [36] (B) RLM-5′ RACE on HvSCL (U21_33945). (C) RLM-5′ RACE on HvSPL (U21_18637). The miRNA binding site is underlined in black and the 5′ end of the cleaved mRNA in red. The numbers in green refer to the ratio of 5′-RACE clones showing a cleavage at the base indicated by the arrow. The agarose gel on the left shows the band corresponding to the amplified 3′ cleavage products. [file 1471-2229-13-6-S3.pdf]

A

```
                                          13.38 RPM
                                          ↓2.87
                                          ↓↓ 9.75
                                          ↓↓  ↓
AK371946 mRNA     5'-CTGGCGCGCGUGAGAUAUUGGCGCGGCUCAAUCACCAGCUUCCCCCC-3'
                                |||||||||:|||||||||||
Hvu-miR171a                  3'-CUAUAACCGUGCCGAGUUAGU-5'

                                             1.84
                                             ↓
AK364580 mRNA     5'-CTGGCGCGCGCGAGAUAUUGGCGCGGCUCAAUCACCGGCUCCCCTCG-3'
                                |||||||||:|||||||||||
Hvu-miR171a                  3'-CUAUAACCGUGCCGAGUUAGU-5'

                                             1.84
                                             ↓
AK368048 mRNA     5'-TCGGCGCGCGCGAGAUAUUGGCACGGCUCAAUCACCGGCUCCCCGCC-3'
                                |||||||||||||||||||||
Hvu-miR171a                  3'-CUAUAACCGUGCCGAGUUAGU-5'

No PARE sequence found

AK362896 mRNA     5'- GCCGGGUGAGUCCGAUGUUGGCAUGGUUCAAUCAAAUUGAGUCGCAA-3'
                                 |||:||||||:||:|||||||
Hvu-miR171a                   3'-CUAUAACCGUGCCGAGUUAGU-5'
```

B

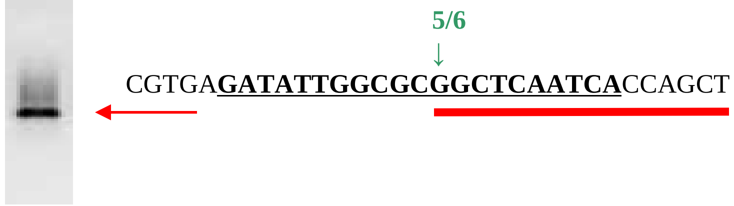

C

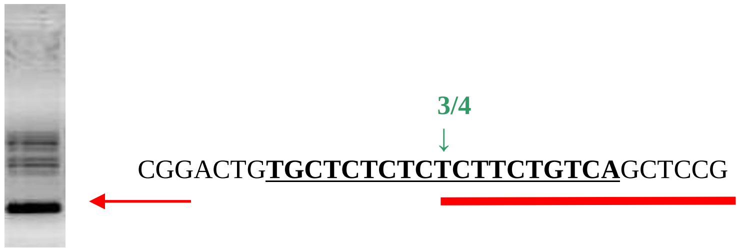

Additional file 3. Evidence for miR171 cleavage of HvSCLs and HvSPL. (A) Evidence for cleavage of top four predicted miR171 targets in degradome data from developing barley grains [36] (B) RLM-5' RACE on HvSCL (U21_33945). (C) RLM-5' RACE on HvSPL (U21_18637). The miRNA binding site is underlined in black and the 5' end of the cleaved mRNA in red. The numbers in green refer to the ratio of 5'-RACE clones showing a cleavage at the base indicated by the arrow. The agarose gel on the left shows the band corresponding to the amplified 3' cleavage products.
