# Supplementary material for: Over-expression of microRNA171 affects phase transitions and floral meristem determinancy in barley
Source: BMC Plant Biol. 2013 Jan 7;13:6. doi: 10.1186/1471-2229-13-6 (PMC3547705; doi:10.1186/1471-2229-13-6)
Supplement: Additional file 4 — Timing of the transition phases during shoot development. The diagram shows the approximate timing of the transitions from juvenile to adult phases and adult to reproductive phases. The first transition was determined by the moment when the first stem of the plant started to elongate (jointing) and the second transition (flowering) when the first spike reached anthesis. Under our SD condition, OE171 plants did not flower. [file 1471-2229-13-6-S4.pdf]

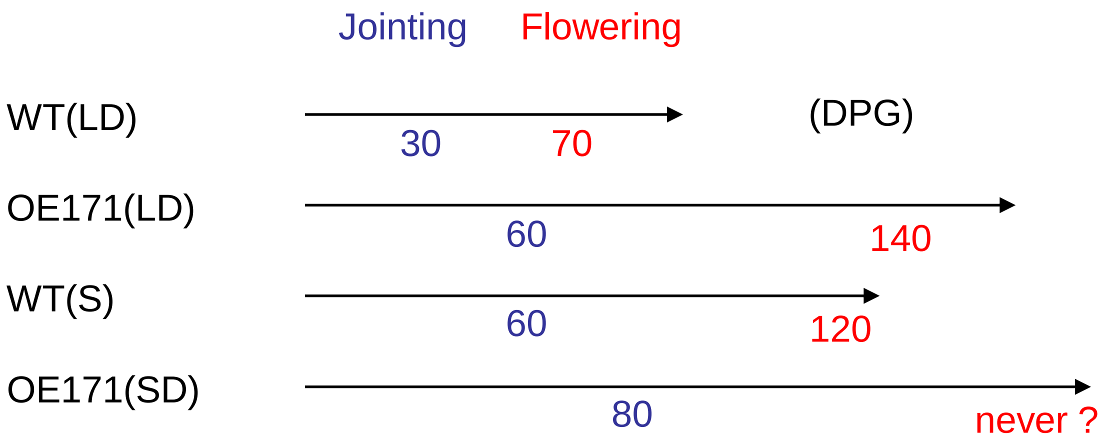

Additional file 4. Timing of the transition phases during shoot development. The diagram shows the approximate timing of the transitions from juvenile to adult phases and adult to reproductive phases. The first transition was determined by the moment when the first stem of the plant started to elongate (jointing) and the second transition (flowering) when the first spike reached anthesis. Under our SD condition, OE171 plants did not flower.
