# Supplementary material for: Over-expression of microRNA171 affects phase transitions and floral meristem determinancy in barley
Source: BMC Plant Biol. 2013 Jan 7;13:6. doi: 10.1186/1471-2229-13-6 (PMC3547705; doi:10.1186/1471-2229-13-6)
Supplement: Additional file 5 — Developmental arrest of OE171-3 (T0). (A) Tiller of the T0 plant OE171-3. Scanning electron microscopy of the SAM of a OE171-3 (B) and WT (C) plants. Ectopic inflorescence meristem (eIM). Scale bars represent 1 mm. [file 1471-2229-13-6-S5.pdf]

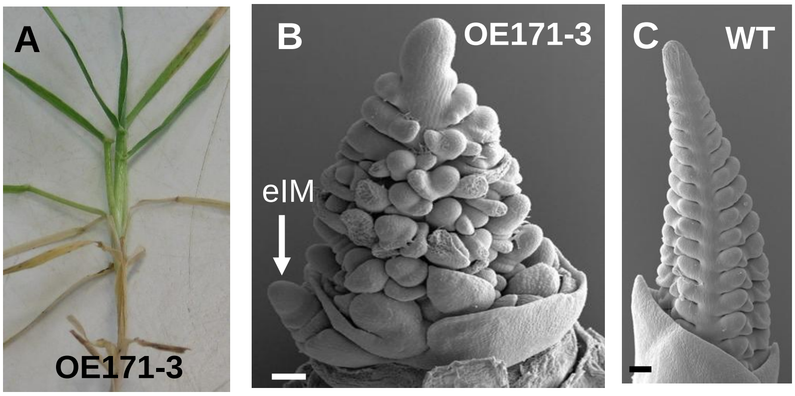

Additional file 5. Developmental arrest of OE171-3 (T0). (A) Tiller of the T_0 plant OE171-3. Scanning electron microscopy of the SAM of a OE171-3 (B) and WT (C) plants. Ectopic inflorescence meristem (eIM). Scale bars represent 1mm.
