# Supplementary material for: Over-expression of microRNA171 affects phase transitions and floral meristem determinancy in barley
Source: BMC Plant Biol. 2013 Jan 7;13:6. doi: 10.1186/1471-2229-13-6 (PMC3547705; doi:10.1186/1471-2229-13-6)
Supplement: Additional file 6 — Comparison of vegetative WT and OE171 plants. Representative WT and OE171 plants at 4 weeks old in LD conditions. [file 1471-2229-13-6-S6.pdf]

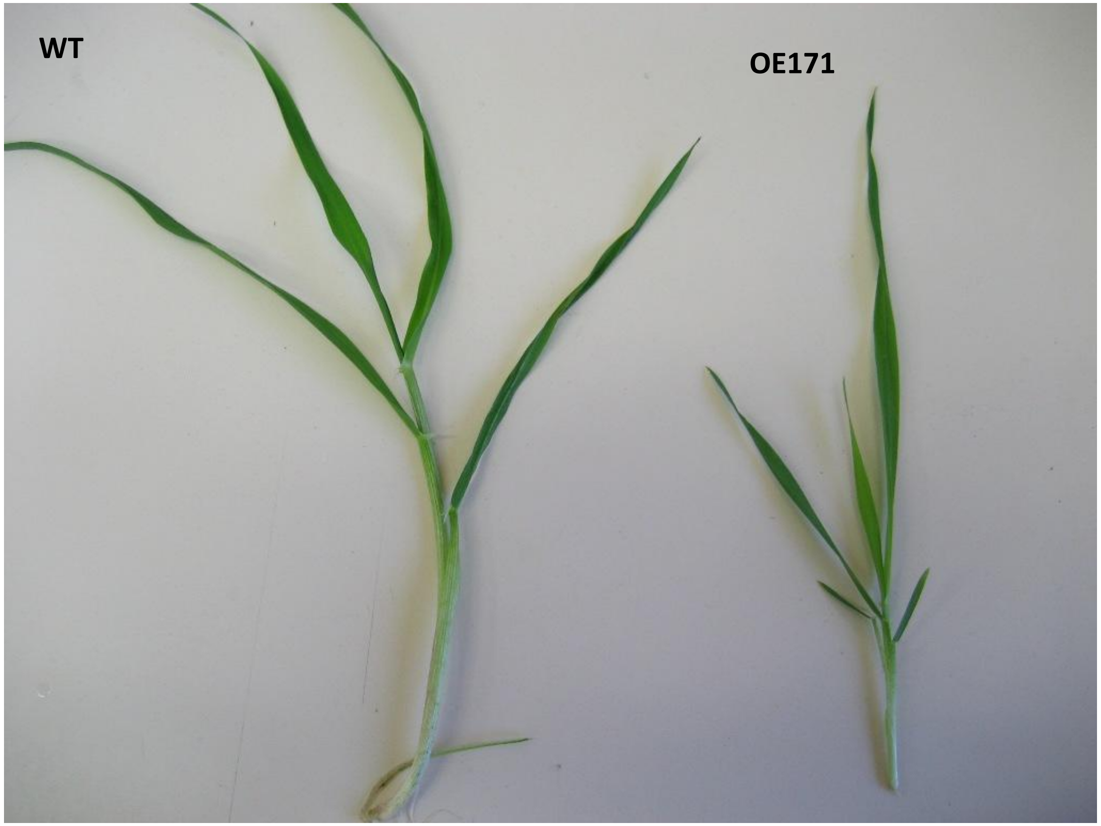

Additional file 6 (pdf file). Comparison of vegetative WT and OE171 plants.
Representative WT and OE171 plants at 4 weeks old in LD conditions.
