# Supplementary material for: Over-expression of microRNA171 affects phase transitions and floral meristem determinancy in barley
Source: BMC Plant Biol. 2013 Jan 7;13:6. doi: 10.1186/1471-2229-13-6 (PMC3547705; doi:10.1186/1471-2229-13-6)
Supplement: Additional file 7 — Primer and Probe sequences used in this study. [file 1471-2229-13-6-S7.pdf]

A. pri-miR171a (U21_38648)

```
           10         20          30          40
U     U          U  A    C    -   A-|     G   GA
 CACUA GAUGUUGGC CG CUCA UCAG ACC   CGCCG AGG  G
 GUGUA CUAUAACCG GC GAGU AGUC UGG   GCGGC UCU  C
C     U          U  C    U    U   CG^     G   AC
 90         80         70         60         50
```

B. Mature miR171 sequences

**hvu-miR171a**	ugauugagccgugccaauauc
**hvu-miR171b**	ugauugagccgcgccaauauc
**ath-miR171a**	ugauugagccgcgccaauauc
ath-miR171b/c	uugagccgugccaauaucacg
**osa-miR171a**	ugauugagccgcgccaauauc
**osa-miR171b-f**	ugauugagccgugccaauauc
osa-miR171g	gaggugagccgagccaauauc
osa-miR171h	gugagccgaaccaauaucacu
osa-miR171i	ggauugagccgcgucaauauc

Additional file 1. miR171 precursor and mature sequences. Information retrieved from miRBase (release-17) and Schreiber *et al.*, 2011 [34]. (A) *pri-miR171a* secondary structure obtained using MFOLD (http://mfold.rna.albany.edu), red letters indicate mature miRNA sequence. (B) mature miR171 sequences found in *Hordeum vulgare* (hvu), Arabidopsis *thaliana* (Ath) and *Oryza sativa* (Osa).

Genbank ID	Homology	Score	Sequence Alignment	Activity
miR171a targets				
AK368048	SCL	0.0	miRNA 21 CUAUAACCGUGCCGAGUUAGU 1<br>:::::::::::::::::::::<br>Target 932 GAUAUUGGCACGGCUCAAUCA 952	CL
AK371946	SCL	0.5	miRNA 21 CUAUAACCGUGCCGAGUUAGU 1<br>:::::::::.:::::::::::<br>Target 1231 GAUAUUGGCGCGGCUCAAUCA 1251	CL
AK364580	SCL	0.5	miRNA 21 CUAUAACCGUGCCGAGUUAGU 1<br>:::::::::.:::::::::::<br>Target 616 GAUAUUGGCGCGGCUCAAUCA 636	CL
AK362896	Unknown function	1.5	miRNA 21 CUAUAACCGUGCCGAGUUAGU 1<br>:::.::::::.::.:::::::<br>Target 446 GAUGUUGGCAUGGUUCAAUCA 466	CL
AK360395	ATPase	4.0	miRNA 21 CUAUAACCGUGCCGAGUUAGU 1<br>::.:::: .: :::::::::<br>Target 926 UAUGUUGGAGCUGCUCAAUCA 946	TR
AK374235	WD40	4.0	miRNA 21 CUAUAACCGUGCCGAGUUAGU 1<br>::. ::::: ::.:::::::<br>Target 959 CAUGAUGGCAAGGUUCAAUCA 979	CL
AK373446	Unknown function	4.0	miRNA 21 CUAUAACCGUGCCGAGUUAGU 1<br>: ::: :: :::::::::::<br>Target 1247 UAGAUUAGCCCGGCUCAAUCA 1267	CL
AK368182	RNA binding protein	4.0	miRNA 21 CUAUAACCGUGCCGAGUUAGU 1<br>::::::::::.: .::. :::<br>Target 893 GAUAUUGGCAUGCUUCGCUCA 913	TR
AK252561	Unknown function	4.0	miRNA 21 CUAUAACCGUGCCGAGUUAGU 1<br>::::::: ::::::.:::.<br>Target 96 UUUAUUGGCCCGGCUCGAUCG 116	CL
miR171b targets				
AK371946	SCL	0.0	miRNA 21 CUAUAACCGCGCCGAGUUAGU 1<br>:::::::::::::::::::::<br>Target 1231 GAUAUUGGCGCGGCUCAAUCA 1251	CL
AK364580	SCL	0.0	miRNA 21 CUAUAACCGCGCCGAGUUAGU 1<br>:::::::::::::::::::::<br>Target 616 GAUAUUGGCGCGGCUCAAUCA 636	CL
AK368048	SCL	1.0	miRNA 21 CUAUAACCGCGCCGAGUUAGU 1<br>::::::::: :::::::::::<br>Target 932 GAUAUUGGCACGGCUCAAUCA 952	CL
AK362896	Unknown function	2.5	miRNA 21 CUAUAACCGCGCCGAGUUAGU 1<br>:::.::::: .::.:::::::<br>Target 446 GAUGUUGGCAUGGUUCAAUCA 466	CL
AK360395	ATPase	3.5	miRNA 21 CUAUAACCGCGCCGAGUUAGU 1<br>::.:::: :: :::::::::<br>Target 926 UAUGUUGGAGCUGCUCAAUCA 946	TR
AK248532	E3 ubiquitin ligase	3.5	miRNA 21 CUAUAACCGCGCCGAGUUAGU 1<br>:::::: : :: ::::.::::<br>Target 164 GAUAUUCGAGCUGCUCGAUCA 184	TR
AK366340	Phosphatase associated protein	4.0	miRNA 21 CUAUAACCGCGCCGAGUUAGU 1<br>:::.::: :: ::::::: ::<br>Target 1890 GAUGUUGACGAGGCUCAAGCA 1910	TR
AK373446	Unknown function	4.0	miRNA 21 CUAUAACCGCGCCGAGUUAGU 1<br>: ::: :: :::::::::::<br>Target 1247 UAGAUUAGCCCGGCUCAAUCA 1267	CL
AK252561	Unknown function	4.0	miRNA 21 CUAUAACCGCGCCGAGUUAGU 1<br>::::::: ::::::.:::.<br>Target 96 UUUAUUGGCCCGGCUCGAUCG 116	CL

Additional File 2. Potential targets of hvu-miR171a and b. psRNAtarget (http://plantgrn.noble.org/psRNATarget) was used to predict potential targets of hvu-miR171a and b among the full-length barley ESTs assembled by Matsumoto et al 2011 [35]. The maximum score was set at 4. The column “Activity” refers to the type of inhibition predicted, Cleavage (CL) or Translation inhibition (TR).

A

```
                                              13.38 RPM
                                              ↓2.87
                                              ↓↓ 9.75
                                              ↓↓ ↓
AK371946 mRNA     5'-CTGGCGCGCGUGAGAUAUUGGCGCGGCUCAAUCACCAGCUUCCCCCC-3'
                                 |||||||||:|||||||||||
Hvu-miR171a                   3'-CUAUAACCGUGCCGAGUUAGU-5'

                                                  1.84
                                                  ↓
AK364580 mRNA     5'-CTGGCGCGCGCGAGAUAUUGGCGCGGCUCAAUCACCGGCUCCCCTCG-3'
                                 |||||||||:|||||||||||
Hvu-miR171a                   3'-CUAUAACCGUGCCGAGUUAGU-5'

                                                  1.84
                                                  ↓
AK368048 mRNA     5'-TCGGCGCGCGCGAGAUAUUGGCACGGCUCAAUCACCGGCUCCCCGCC-3'
                                 |||||||||||||||||||||
Hvu-miR171a                   3'-CUAUAACCGUGCCGAGUUAGU-5'

No PARE sequence found

AK362896 mRNA     5'- GCCGGGUGAGUCCGAUGUUGGCAUGGUUCAAUCAAAUUGAGUCGCAA-3'
                                  |||:||||||:||:|||||||
Hvu-miR171a                    3'-CUAUAACCGUGCCGAGUUAGU-5'
```

B

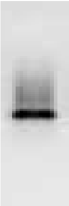

5/6
↓
CGTGA**<u>GATATTGGCGCGGCTCAATCA</u>**CCAGCT

C

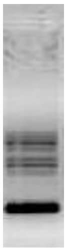

3/4
↓
CGGACTG**<u>TGCTCTCTCTCTTCTGTCA</u>**GCTCCG

Additional file 3. Evidence for miR171 cleavage of HvSCLs and HvSPL. (A) Evidence for cleavage of top four predicted miR171 targets in degradome data from developing barley grains [36] (B) RLM-5' RACE on HvSCL (U21_33945). (C) RLM-5' RACE on HvSPL (U21_18637). The miRNA binding site is underlined in black and the 5' end of the cleaved mRNA in red. The numbers in green refer to the ratio of 5'-RACE clones showing a cleavage at the base indicated by the arrow. The agarose gel on the left shows the band corresponding to the amplified 3' cleavage products.

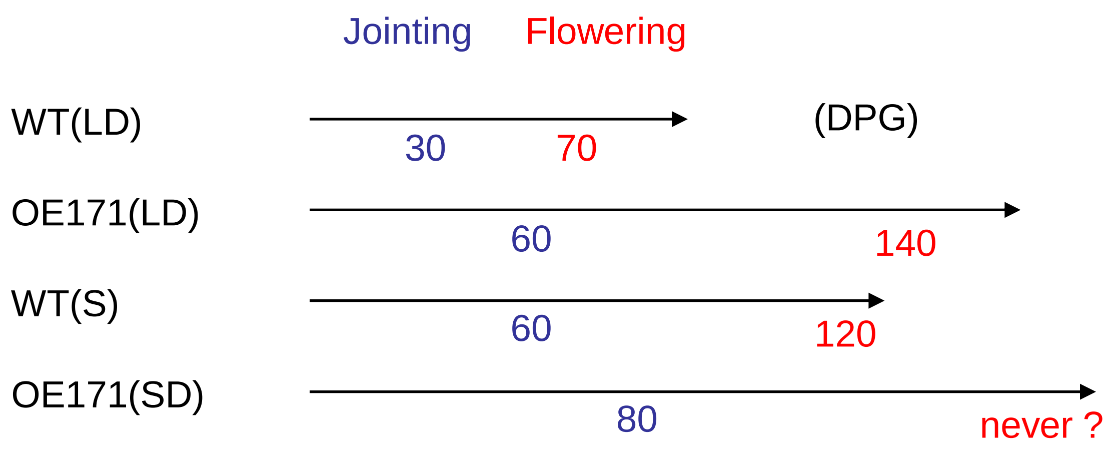

Additional file 4. Timing of the transition phases during shoot development. The diagram shows the approximate timing of the transitions from juvenile to adult phases and adult to reproductive phases. The first transition was determined by the moment when the first stem of the plant started to elongate (jointing) and the second transition (flowering) when the first spike reached anthesis. Under our SD condition, OE171 plants did not flower.

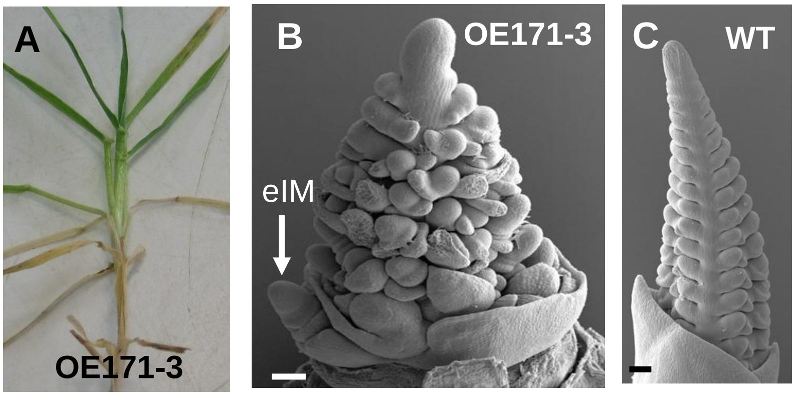

Additional file 5. Developmental arrest of OE171-3 (T0). (A) Tiller of the T_0 plant OE171-3. Scanning electron microscopy of the SAM of a OE171-3 (B) and WT (C) plants. Ectopic inflorescence meristem (eIM). Scale bars represent 1mm.

Name	Sequence 5’-3’	Use for
MIR171-1F	CACCTTGGTGCTAGAGGCTAGAGAG	Cloning
MIR171-2R	GATCCGATGATAAAACCCCTTCG	Cloning
MIR171-3F	CGGCGGCGGTTCTGATTG	qPCR
MIR171-4R	GCGTGCATGAAAGAGCACTAA	qPCR
SCL-F	GCGGCTTACAAGTCCTTCTC	qPCR
SCL-R	GAAGTTAGCGAACTGCAGCAT	qPCR
SPL-F	TTCTCCGATGGTCTGACTCC	qPCR
SPL-R	ATTGCTGCAGGTTGGAGAAC	qPCR
AP2L-F	TGCAGCCAGAGATCATAGCA	qPCR
AP2L-R	GGTTGCTTGACGACGATGAT	qPCR
TRD-F	TTAAATAACAGAGCGCCGAGA	qPCR
TRD-R	GCTGTGGTCGTCGTAGGAGTA	qPCR
PLA1-F	GCTCTGGGAGATGGTGTTCC	qPCR
PLA1-R	GATGACGGCGTGGAGGTAG	qPCR
WUS-F	ACCTTTTGTCTCTCCCTCCAC	qPCR
WUS-R	ACATCTCCTCCAGGATCATCA	qPCR
KN1-F	TCAAGGAGATGCTGCTCAAGA	qPCR
KN1-R	GTAGTGCGTGTTCCACCAGTC	qPCR
miR171-AS	GATATTGGCACGGCTCAATCA	Northern Probe
miR156-AS	GTGCTCACTCTCTTCTGTCA	Northern Probe
miR172-AS	ATGCAGCATCATCAAGATTCT	Northern Probe
miR168-AS	GTCCCGATCTGCACCAAGCGA	Northern Probe
SCL-5RACE	GAGAAGGACTTGTAAGCCGCC	5’RACE
SPL-5RACE	GAGACGGTGCCGGTGGAAGCCT	5’RACE

Additional file 7. Primer and Probe sequences used in this study.
